# Financial production and the subprime mortgage crisis

**DOI:** 10.1007/s00191-023-00812-y

**Published:** 2023-03-13

**Authors:** Daniele Tori, Eugenio Caverzasi, Mauro Gallegati

**Affiliations:** 1 The Open University Business School, Milton Keynes, United Kingdom; 2 Università degli Studi dell’Insubria, Varese, Italy; 3 Università Politecnica delle Marche, Ancona, Italy

**Keywords:** Financial firms, Innovation, Crisis, Securitization, **s**: B52, E44, G21

## Abstract

The causes of the 2007-8 subprime crisis continue to be the subject of much debate, with explanations ranging from de-regulation and fraudulent behavior to global imbalances and rising inequality. However, a comprehensive analysis of the endogenous forces that made the crisis inevitable has yet to be presented. This paper offers a ‘structural’ interpretation of the crisis by synthesising insights from conventional financial economics and the Minskyian and Schumpeterian literature. While highlighting the innovative character of US financial firms evolving from credit providers to *producers* of financial commodities, we stress the key features of their path towards financial fragility.  We contend that financial institutions were able to achieve progressively unsustainable positions due to the ‘enforced indebtedness’ of US households, which played a functional, albeit secondary, role in the development of the crisis.

## Introduction

The assessment of the causes of crises in capitalist systems is one of the core topics in economic analysis. Debates about the major financial crisis from the XVIII century are still open today. The Global Financial Crisis (henceforth GFC) of 2007-8 makes no exception. In his review of twenty-one books on this crisis, Lo (2012:151) argues that “no single narrative emerges from this broad and often contradictory collection of interpretations”, suggesting a situation of ‘multiple truths’.^1^

The general view seems to be that the crisis resulted from an interaction of various aspects related to regulation (Reinhart and Rogoff 2008; Baker 2010), loose monetary policy (Alessi and Detken 2011), and irrational behaviours (Shiller 2015), which generated an insolvency/counterparty risk crisis (Thakor 2015). Rather than a single factor triggering the catastrophic event, a complex interconnection between several components seems to be the emerging picture.

The mainstream argument is well summarized by Brunnermeier (2009). Large capital inflow, especially from Asian countries willing to hedge local currencies against the dollar, resulted in the US adopting a low-interest rates monetary policy to prevent deflation. Meanwhile, innovation in the financial sector through securitization enhanced the supply of securities (and liquidity), mainly built on fixed-income instruments, namely mortgages. The huge growth in securitization led to “an opaque web of interconnected obligations” (Brunnermeier 2009:98) which ended in the massive liquidity crisis when house prices sharply declined.

Scholars focusing on the importance of global imbalances argue that the period of strong growth before the crisis featured widening current account deficits in advanced economies (mainly the US) and growing current account surpluses in commodity exporters countries (mainly China). The high liquidity in the global financial markets was fuelled by the substantial and lasting easing of US monetary policy and the anchoring to the US dollar of emerging markets’ exchange rate (Ackerman, 2008). Too large imbalances carry the risk of substantial adjustments in exchange rates and asset prices, hence damaging growth prospects. Global imbalances are the result of various factors linked to the increase in international capital flows and their negative effects on global interest rates, hence fostering the credit boom (Astley et al. 2009).

An alternative body of literature focuses on income distribution and financial instability as key aspects to explain the crisis. On the one hand, a ‘microeconomic’ view argues that rising inequality since the 1980s caused a reduction in households’ saving rates and increasing debt, thus stimulating a credit bubble (see among others Stiglitz, 2009; Rajan, 2010).^2^ On the other hand, the growth of innovative ways of financing would not have been possible “without investors’ strong demand for high-margin, higher-risk assets” (Ackermann 2008:330). Lysandrou (2011), Goda and Lysandrou (2014), and Lysandrou and Nesvetailova (2015) show how global wealth concentration had a central role in nurturing the demand for structured financial products (e.g., ABSs and CDOs). Accordingly, inequality is presented as the key element to explain investors’ demand for high-yielding assets. However, Stockhammer (2015) stresses that the interactions between inequality and financial innovation need to be studied further. Income inequality plays a role also in some Marxian explanations of the GFC. To summarize, according to these authors, stagnant real wages and increasing profits from the late 1970s resulted in a potential investment realization problem, resolved through increases in indebtedness, net exports, and speculation (see among others Foster and Magdoff, 2009; Kotz, 2009).

From a broader theoretical perspective, numerous authors stressed the inadequacy of the economic mainstream in face of the crisis (e.g., Kirman, 2010). The confidence in the (financial) markets’ self-regulating ability mirrored the lack of a theory of financial crises.^3^ A theory of financial instability ought to be found elsewhere. Indeed, since the 2007-8 financial crisis, Hyman Minsky’s theories have had a huge comeback, giving rise to an alternative reading of the GFC. Aiming to provide a theoretical ground for the understanding of the recent financial crisis, his Financial Instability Hypothesis (Minsky, 1986, henceforth FIH) has abundantly been referred to by various authors (e.g., Eggertsson and Krugman, 2012; Bhattacharya et al. 2015). The FIH can be defined as an endogenous theory of the business cycle based on the analysis of the financial structure of the economy (more on this in Section 3). The economic system is characterized by endemic forces that during a period of tranquillity - meant as a situation of stable growth - lead the system towards instability: “*stability… is destabilizing*” (Minsky 1975). The term ‘Minsky Moment’ has become a catchy expression to define a situation in which the indebtedness of some sector of the economy becomes unsustainable, ultimately triggering an economic crisis.

If the original FIH referred to the firm sector, some studies put forward what we could define as a ‘same scenario different location’ perspective, according to which a ‘Minsky moment’ actually occurred, albeit not exactly *where* Minsky theorized it would have happened - i.e., the traditional businesses sector - but within households (e.g., Dymski 2009; Bellofiore and Halevi 2009). However, the application of Minsky’s FIH to the *subprime* crisis bears its difficulties, to which we will return later.

The broadly mainstream and alternative literature provide useful insights into the different mechanisms beyond the build-up of the 2007-8 crisis. Notwithstanding their theoretical richness, almost 15 years after the crisis, the debate about its main causes is still wide open.

Ultimately, what different analyses identify as key driving forces of the crises depends on the theoretical lens employed. In an attempt to reconcile the abundance of interpretations with the broader theoretical debate, Skidelsky (2009) identifies two main macroeconomic explanations. In a nutshell, the first, linked to monetarist theory, blames the loose monetary policy for having ignited the credit boom which led to the crisis. For the second - the Keynesian or ‘saving glut’ thesis in the words of Skidelsky – the cause of the crisis lays in the lack of aggregate investment with respect to saving: too few (productive) assets were created^4^, driving the private sector towards over-indebtedness. According to Skidelsky, in the monetarist explanation, the causal link runs from financial markets to real sectors, while the opposite holds for Keynesians. Although useful, this classification bears non-trivial difficulties. In our view, (i) stock and flow dynamics are not clearly distinct, and (ii) the identification of the Keynesian explanation with a unidirectional real to financial-sector dynamics is too simplistic. Concerning the former, we believe that portfolio choices on the stock of savings of domestic or foreign actors played a role in the starting phases of the crisis. However, the credit and asset price booms appear to be ultimately due to the ‘excess elasticity’ of the global monetary and financial system (Borio and Disyatat 2010). This perspective, in line with post-Keynesian monetary theory (Lavoie 2016), shifts focus from stocks (savings) to flows (credit creation). As to the latter, the upsurge of financial investments with respect to real ones is indeed central in Keynesian literature, particularly within the contributions on financialization (see among others Krippner, 2005; Tori and Onaran, 2020). However, focusing on a causal nexus unilaterally going from the ‘real’ to the ‘financial’ might wrongly seem to imply a passive role of the financial sector in Keynesian literature.

Notwithstanding the heterogeneity of the approaches, the key aim is to explain the exceptionally high and unsustainable level of household sector debt. We argue that (i) the fragility of the financial position of sectors within the financial system (what we will label *‘financial firms’*) played a central role; (ii) households’ indebtedness largely resulted from dynamic forces taking place within the financial system. The main contribution of this paper is to provide an interpretation of the Global Financial Crisis (GFC) which, albeit encompassing various elements put forward by the literature, is characterized by three fundamental aspects of novelty. First, we use a macro-finance perspective. We thus place financial dynamic forces at the centre of the stage and interpret them in relation to the macroeconomic structure of the economy, also assessing the role of different financial segments within the financial system (see Section 3). Second, we identify as the heart of the crisis what we will label as a process of ‘financial production’. In our view, the production of financial assets to satisfy the appetite of financial investors was the cornerstone around which the unfolding of the endogenous dynamics, which led to the crisis, took place. Third, we develop a theoretical framework combining elements of Minsky and Schumpeter under an evolutionary lens. The evolving nature of the financial system is fundamental to our explanation of the events leading up to the crisis. We emphasize the innovative nature of US bank and non-bank financial institutions, and their evolution into not just intermediaries 2.0 but *creators* of *financial commodities* and *producers* of *financial assets.* We argue that, following a dynamic that can be interpreted under the Minskyian lens, these *financial firms* have been able to reach increasingly unsustainable positions also thanks to US households’ ‘enforced indebtedness’, which played a functional but secondary role in the unfolding of the crisis. We believe this perspective offers a starting point for a ‘structural’ understanding of the crisis, which may go beyond Skidelsky’s dichotomy.

The rest of the paper is structured as follows. Section 2 presents the theoretical foundations of our evolutionary analysis by discussing financial innovations in the context of Minskyian and Schumpeterian contributions. Section 3 presents an empirical assessment of the major trends within the US financial system through a novel analysis of the Financial Accounts of the United States. Section 4 discusses the process of financial production, considering our theoretical foundations and empirical evidence. Section 5 presents our structural reading of the GFC. Section 6 concludes.

## Financial innovations through minskian and schumpeterian lens

The core argument of the mainstream literature is built on the premises of banking and financial sectors as ‘innovative’ actors within ‘intermediation chains’ that, throughout securitization, fuelled the credit bubble leading to the insolvency crisis (Mian and Sufi 2009; Adrian and Ashcraft 2016).^5^ In contrast, we argue that, drawing on Schumpeterian and Minskyian insights, the economic role of financial firms is more complex. 

Schumpeter (1964/1939) describes economic development as a dynamic combination of endogenous determinants (i.e. innovations) and response mechanisms (i.e. business cycles). Entrepreneurial activity is the only process through which new ‘combinations’ of means of production (i.e. innovations) are introduced, thus breaking the otherwise static ‘routine’ (Schumpeter, 1982). The Austrian scholar also made connections between credit and innovation, specifically the provision of credit and the dynamism of economic systems. Entrepreneurs’ appetite for future profits is the key driver of innovative processes. The introduction of innovation makes the appropriation of profits possible, thus enhancing a new phase of accumulation. Schumpeter (2014/1970) understands money as the clearing tool through which credit and debt positions can cancel each other out. By modelling the economy as a system of accounts (households, firms, commercial banks, and the central bank) he highlights the role of the banking system as the sector of the economy in which the account settlement takes place. This gives to banks their unique ability to create new purchasing power, since “*every bank credit and every bank investment creates a deposit*” (Schumpeter, 2014/1970:188, italic in the original). For Schumpeter (1951:153), thanks to credit creation, banks are pivotal players in the innovation process. The process of introducing new combinations cannot be financed through past returns (in the equilibrium of the ‘circular flow’ profits and savings are assumed to be zero, and resources fully utilized), thus requiring fresh credit. In short, innovations would simply be “impossible without new general purchasing power.” (Bellofiore 1985:26). Schumpeter (2014/1970:316) characterizes the operation of the money market as basically “financing of production, trade, and speculation, the transactions of which ultimately require a special financing operation”.

The financial side of the innovation processes is seldom central in the neo-Schumpeterian literature, which instead largely focuses on the ‘real side’ (Block et al. 2017). The work of Perez (2002; 2011) is a praiseworthy exception.^6^ Perez argues that the common traits in all technological revolutions^7^ have been an initial ‘irruption’ stage during which new technologies appear, followed by an injection of funds from financial institutions as a necessary condition for the establishment of a new techno-economic paradigm. In short, the argument is that each major financial bubble was driven by massive processes of credit creation to install each technological revolution (Perez 2002).

Perez’s description of the financial aspects of the neo-Schumpeterian innovation-based business cycle theory recalls both the financial theory of the business cycle and the Financial Instability Hypothesis (FIH) of Minsky (1986).^8^ Minsky’s theory could indeed provide an explanation for a financial crisis born in a period of tranquillity in which borrowers and lenders became progressively reckless. While for Schumpeter (1964/1939:111) credit creation is the “monetary complement of innovation”, Minsky, interpreting the banking and the innovative financial sectors as complementary to traditional entrepreneurial activities, suggests that “Nowhere is evolution, change and Schumpeterian entrepreneurship more evident than in banking and finance” (Minsky, 1993:106). There are nonetheless non-trivial differences among the theories of Minsky and Perez. First, innovations in the real sector are not the driving force in the FIH. Second, Minsky, in line with the aforementioned literature on credit creation (Borio and Disyatat 2010; Lavoie 2016) centred his analysis around the liability side of firms’ balance sheets, hence the inflow of money from the banking sector. Perez refers instead to capital gains (bubbles), and therefore to companies’ stocks (financial markets) rather than commercial banks. Kregel (2009) suggests that Perez’s interpretation does not fully grasp the complexity and evolving nature of the financial system.

Conversely, as steps have been taken in the application of neo-Schumpeterian insights on the financial system, Minsky’s financial interpretation of capitalism offers important intuitions for the analysis of the economy as a system in evolution (Knell 2015). Nonetheless, for sake of clarity, it is important to recall the main features of the FIH. In its traditional version, this theory maintains that firms, under uncertainty, choose the level of investment by comparing two elements in Minsky’s two-price theory (i.e. borrower’s and lender’s risk). The former is the expected stream of returns discounted by a discretionary margin, or a ‘cushion of safety’ (Kregel 1997) to protect firms against the possibility of wrong forecasting and of inability to repay debt (borrower’s risk). The latter is the cost of the external funding and the amount of the loans since banks claim a premium against the possibility that the borrower defaults (lender’s risk). The bigger the loan, the higher the probability of default and subsequent losses, and therefore the risk premium. If revenues exceed commitments, the economy is stable and expanding. Different financial positions emerge, which Minsky labelled *hedge*, *speculative*, and *Ponzi*. In the first case, firms expect that money inflows always exceed their financial commitments (the principal plus debt service). In the second case, their revenues cover interests but are not sufficient to repay the principal and it becomes necessary to roll over the debt. Finally, Ponzi units need to borrow further money or sell assets, since expected revenues are lower than their interest payments alone. After a prolonged period of tranquillity firms’ and banks’ expectations tend to become more and more optimistic. The margins of safety narrow, and speculative and Ponzi finance become consuetudinary. The economic system endogenously moves towards an unstable financial position and then ‘something’ happens. This could be an increase in interest rate - due to tighter monetary policy or endogenous forces - or a change in expectations. At this point, speculative and Ponzi units cannot meet their financial commitments. The upward phase of the cycle comes to an end, an economic crisis is ultimately triggered, and the downward phase of the cycle starts as a Fisherian debt deflation takes place.

In this context, the innovative features of financial institutions are mostly reflected in their ability to circumvent regulations through liability management, finding “new ways to finance activities” (Minsky, 1986:220), thus increasing the supply of credit. Despite the crucial importance of finance within Minsky’s analysis, in his theory, the financing needs are primarily driven by investment decisions in the real sector. We believe that a radical change of perspective is needed to grasp the extent of the impact of recent financial innovations.

Although Minsky was able to identify the emergence of a new stage of capitalism, i.e. money manager capitalism (MMC), he did not have the opportunity to observe its unfolding. The financial system has experienced a ‘technological revolution’ that characterizes a new regime of accumulation, with a ‘cluster of innovations’ that can be placed under the label ‘securitization’. The evolution experienced by financial institutions led them to transcend their traditional role as credit providers, making them ‘producers’ of financial commodities. This double function was noted by Schumpeter himself (1951:153, footnote 8): “the introduction of new banking practices may constitute enterprise, and bankers (or other ‘financiers’) may use the means at their command in order to embark upon commercial and industrial enterprise themselves.”

Interpreting Minsky’s contribution as an adaptable framework allows us to understand how the financial system can overcome its limits. In our view, the FIH is indeed the outcome of Minsky’s ‘financial Keynesianism’, a complex theoretical framework stemming from his personal interpretation that highlights the ‘hidden’ financial aspects of Keynes’ General Theory. Some of the key theoretical elements of his analysis are standard components of the post-Keynesian framework. Examples are fundamental uncertainty, money endogeneity, the principle of effective demand, the key role of investment and the macroeconomic determination of profit, and the ‘monetary interpretation’ of capitalism. Other elements are more distinctive of Minsky’s work.^9^ Above all is the centrality of finance - “a capitalist […] economy is a financial system” (Minsky, 1992:16). This should be seen as appreciating the balance sheet implications of economic decisions at the macroeconomic level. Each investment decision implies a choice on the structure of the liability side of a balance sheet. There is an inner inter-temporality attached to agents’ choices, for instance, current investment decisions validate (through the determination of aggregate profits) debt positions taken in the past and determine future debt positions. Balance sheets are highly interconnected, and this leads to the propagation of financial distress. In his view, “the missing step in the standard Keynesian theory was the explicit consideration of capitalist finance within a cyclical and speculative context.” (Minsky 1975:129). Finally, Minsky considered capitalism to be a system in continuous evolution, whose different stages, at times coexisting, follow one another. The stages differ with respect to the features of trades and industries, production costs, and balance of powers. Minsky (1988) characterizes these phases mostly according to the role and features of financial institutions. The last stage he identified, namely Money Manager Capitalism sees the rise of institutional investors: the development of private pensions led to a massive accumulation of wealth in the hands of pension funds, while the money market and mutual funds emerged as a more convenient alternative to savings and deposits account due to inflation and regulations. A large portion of corporate shares and bonds is therefore owned by funds, whose short-termed vision due to the need to remunerate investors, influences corporate management.^10^ MMC represents a further step in the process of detachment of corporations’ financial needs from financial intermediaries toward capital markets, which resulted in banks changing their business becoming focused more on fee income-related activities and providing finance to other financial institutions. A further feature of MMC is the rise of securitization, which deeply changed the face of the financial system and is central in this new phase of capitalism: ‘There is a symbiotic relationship between the growth of securitization and money managed capitalism’ (Minsky 1988:36). He recognised the ability of securitization to allow overcoming balance sheet limitations and to transfer the risk to the holder of the securities.

The evolutionary interpretation of capitalism put forward by Minsky himself motivates our attempt to provide a new reading of the FIH. Minsky (1993:106) argued that a prerequisite to grasping the dynamics of the business cycle and the structural evolution of economic systems is the understanding of “the financing relations that rule, and how the profit-seeking activities of businessmen, bankers, and portfolio managers lead to the evolution of financial structures.” For this reason, it can be argued that the FIH is just as much about economic evolution as it is about effective demand in a monetary economy (Knell 2015). Moreover, relying on a slavish reading of the FIH to explain the GFC introduces some difficulties: neither the crisis can be simply read as the result of excessive loans granted to traditional firms to finance their investment decisions, nor the taxonomy of financial position applies to the households’ sector without due adaptations (Pressman and Scott 2018). This raised some controversies about the extent to which the FIH can explain the so-called ‘sub-prime crisis’ (e.g., Davidson, 2008; Behlul, 2014). In our opinion, the FIH should be understood indeed as a corollary of a broader theoretical framework, ultimately aimed at describing the macro-dynamic features of financially complex market economies. Therefore, it should be updated to consider the evolution of the financial sector system. In line with Variato (2015), we recognize both the inability of the original FIH to exactly describe the *subprime* crisis, and the usefulness of applying Minsky’s financial-Keynesian theoretical framework to analyze contemporary financial markets, hence obtaining a new reading of the FIH and a structural explanation of the recent financial crisis. The data presented in the next section aims to provide descriptive empirical support for a ‘financial version’ of the FIH.

## An empirical assessment

**Fig. 1:**
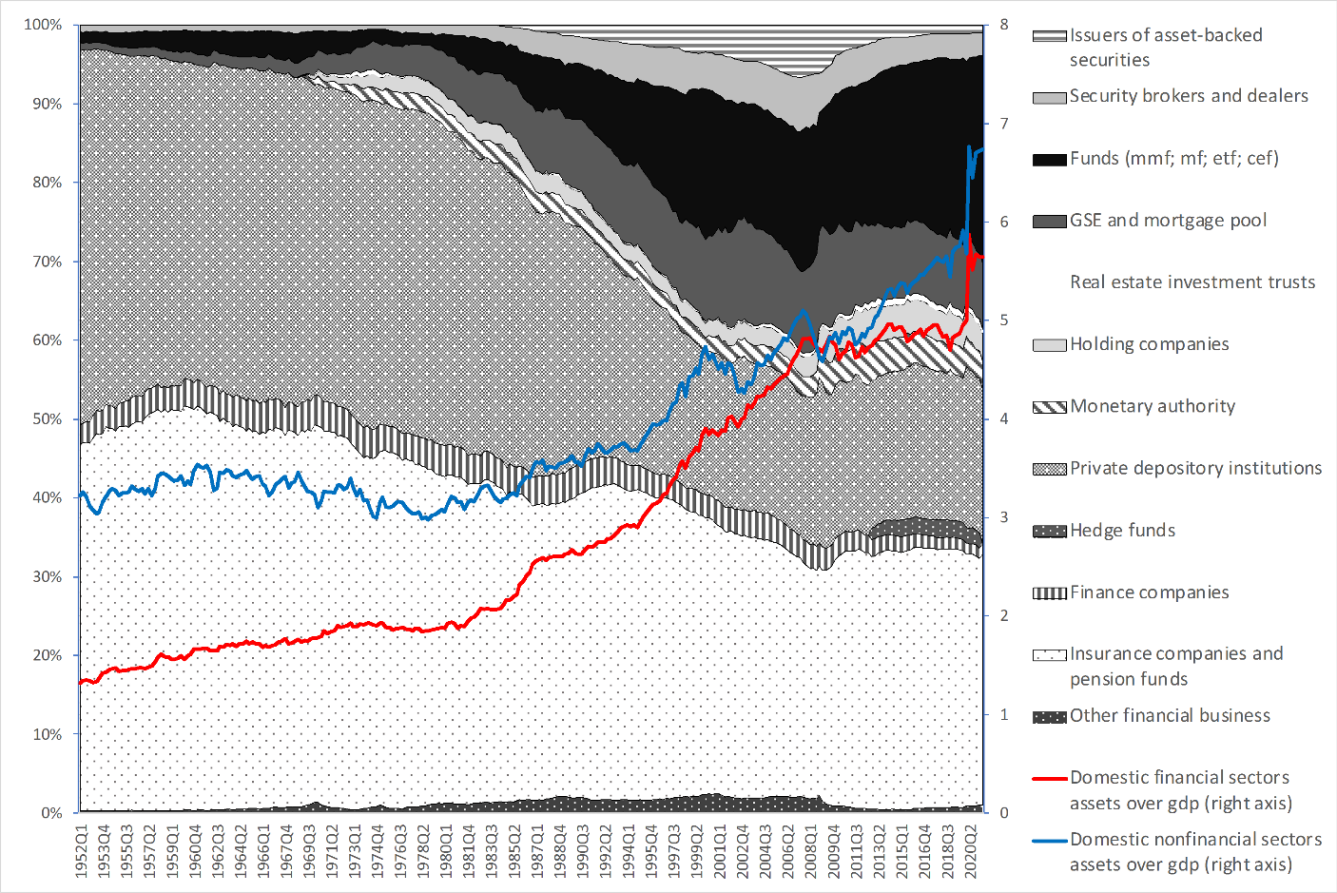
US domestic financial system composition; source: Financial Accounts of the United States, Z.1 flow of funds

**Fig. 2:**
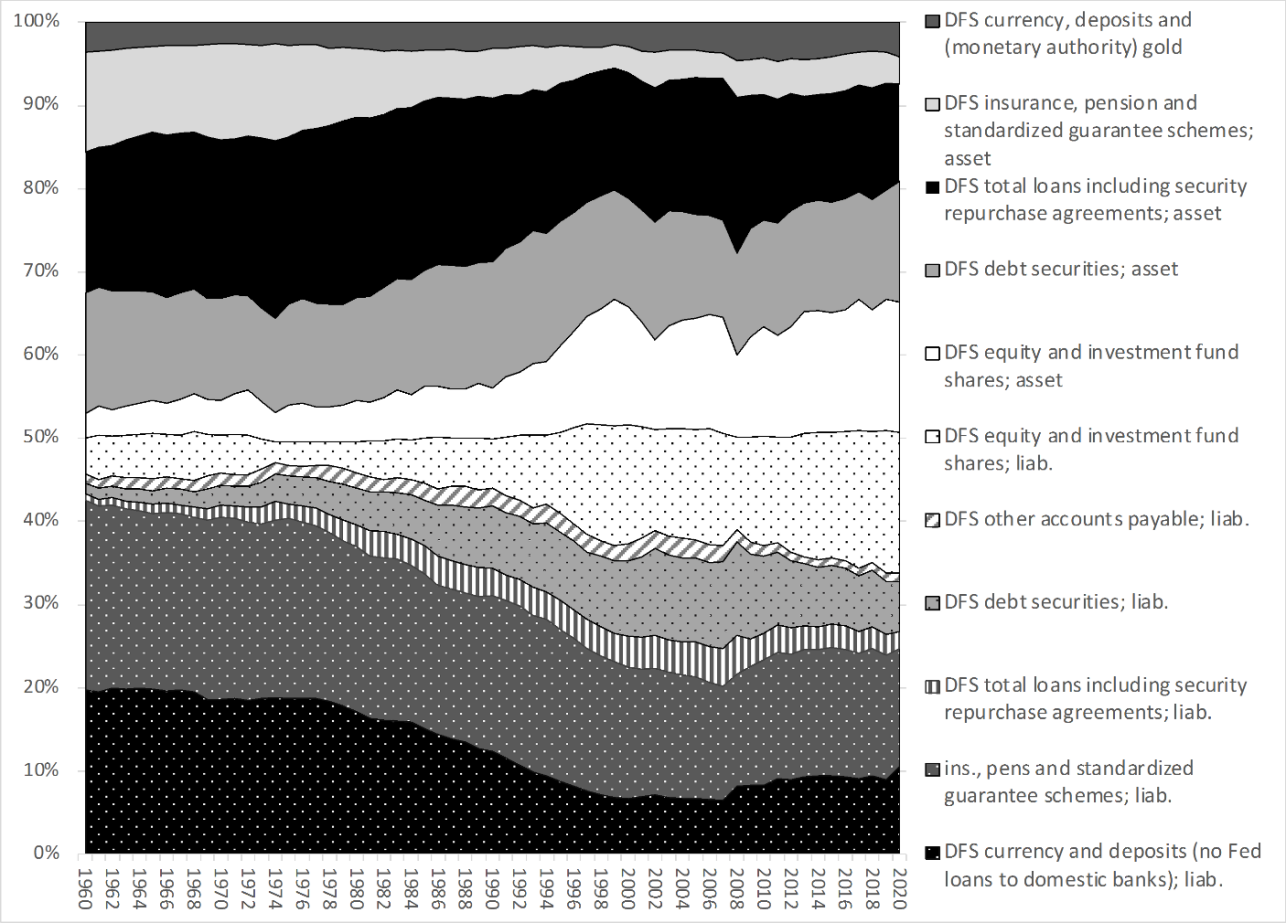
US domestic financial sector aggregate balance sheets (liabilities patterned fill); source: Financial Accounts of the United States, Z.1 flow of funds

**Fig. 3:**
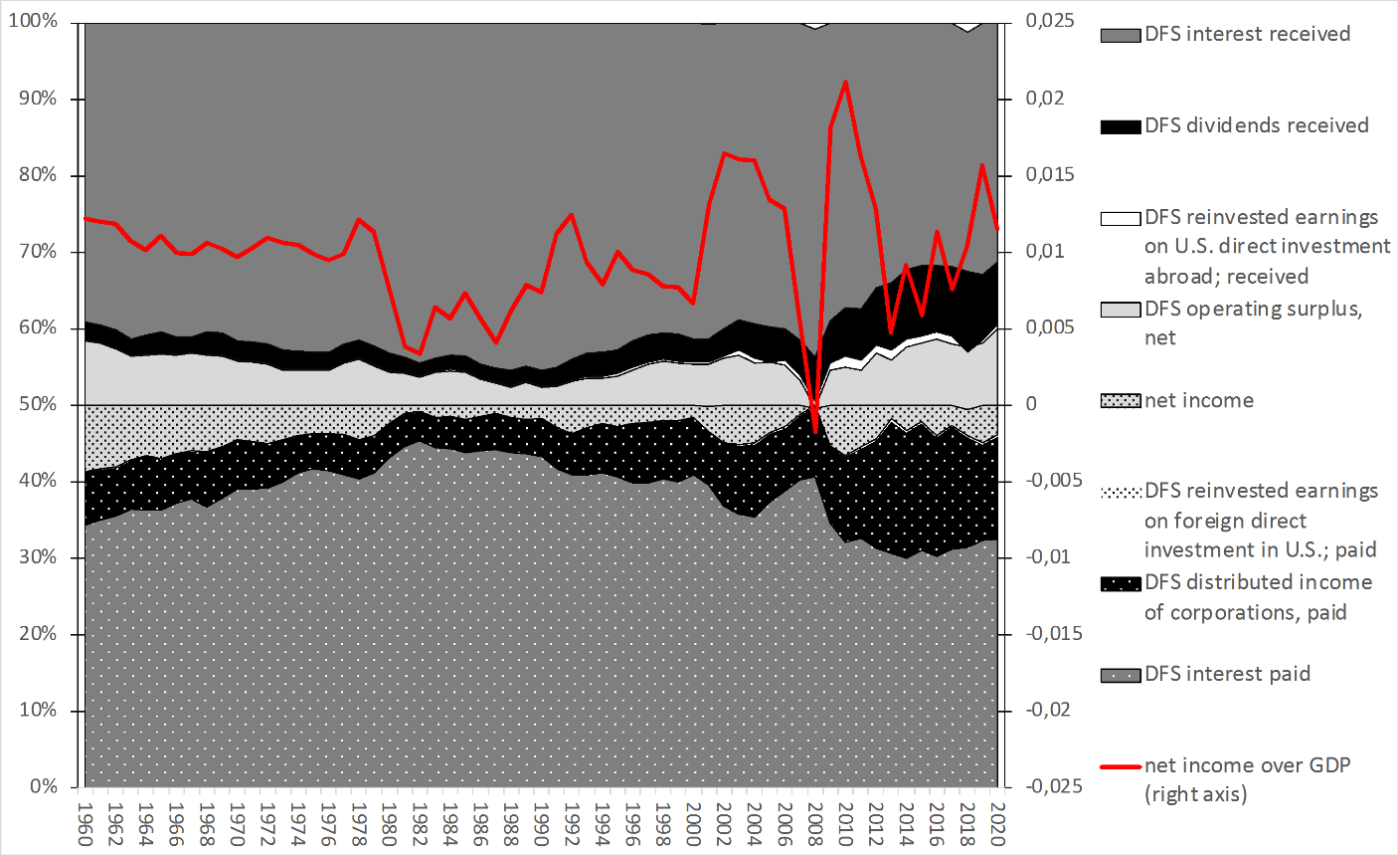
US domestic financial sector flows (outflows patterned fill); source: Financial Accounts of the United States, Z.1 flow of funds

**Fig. 4:**
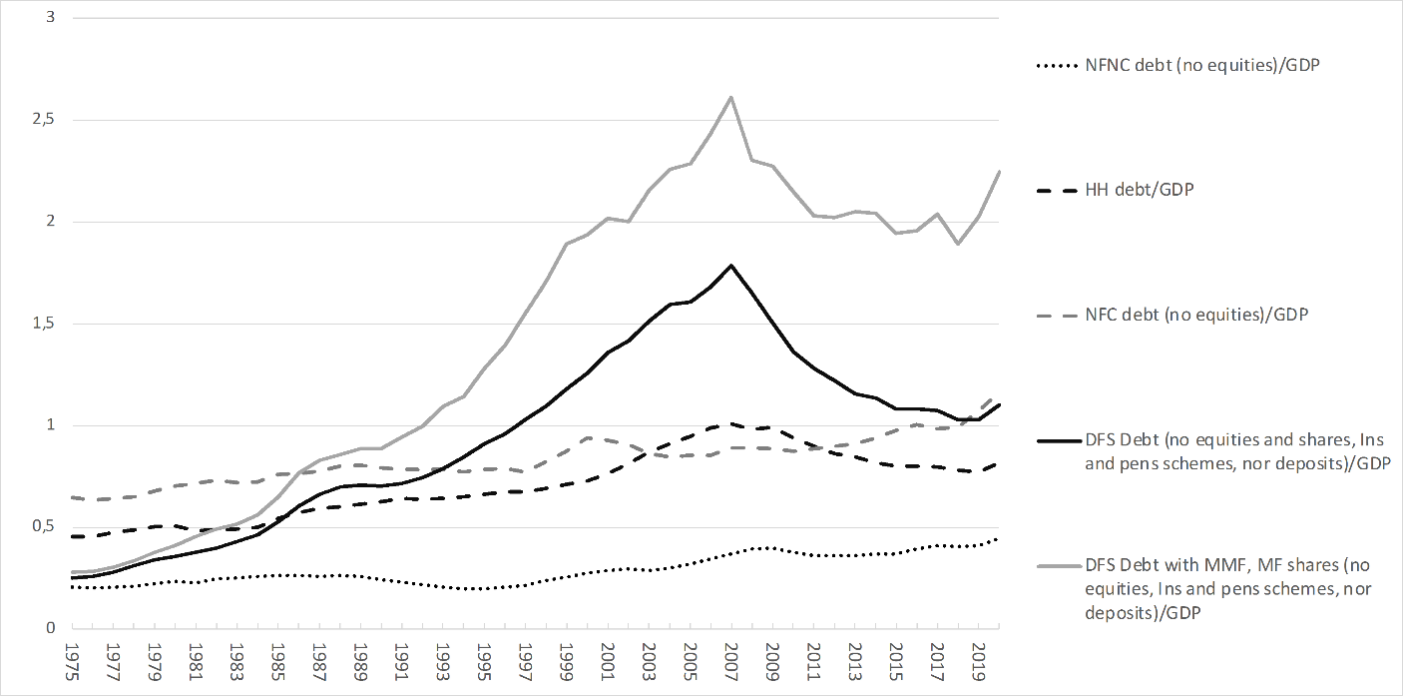
US debt to GDP ratios, main sectors; source: Financial Accounts of the United States, Z.1 flow of funds

This section presents and discusses some key stylized facts about the major trends in the US financial system from the 1970s. First, we look at the financial system (FS) to assess its evolution in terms of (i) sectoral composition (Fig. 1), (ii) stocks of both assets and liabilities (Fig. 2), (iii) inflows and outflows (Fig. 3), and (iv) debt to income ratios (Fig. 4). We then try to offer a more granular perspective, analysing those components of the financial sectors in which the outburst of the crisis took place.

The GFC was systemic in nature, as it was linked to the evolution of the entire FS, and it transcended the domestic dimension due to the worldwide centrality of the US monetary system (CGFS 2020). However, just like earthquakes have a hypocentre (where they originate in the underground) and an epicentre (the corresponding point on the surface), the crisis had its hypocentre in the securitising system and, stretching a bit the similitude, its epicentre in the *subprime* mortgage market.

Figure 1 shows the key transformation of the FS in terms of the types of financial institutions involved.^11^ Traditional sectors, such as private depository institutions, insurance companies, and pension funds dramatically decreased their relative sizes. The counterpart is the boom of different types of funds (Money Market Funds, Mutual Funds, Exchange Traded Funds, and Closed-End Funds) together with the rise of the GSE-Mortgage Pool, Issuers of Asset-Backed Securities, and Security Brokers and Dealers. These were either minor or absent until the 1980s, with a combined weight of about 10% of the whole financial sector; at the outburst of the crisis, they had grown up to around 40%. In particular, the funds alone represented 20% of the FS, and after a few years of decline, they represent around one-fourth of it. Issuers of asset-backed securities saw their weight dramatically decrease from 7% to less than 1%.

This metamorphosis was associated with a rapid expansion of the FS vis-à-vis the non-financial sector, as shown in Fig. 1 by the ratio of financial assets over GDP of the FS (red line) chasing and (briefly) overcoming those of the non-financial sector (blue line) in correspondence of the crisis. Both measured just below five times the GDP.

The transformation can be appreciated also in terms of the overall balance sheet composition (Fig. 2). Concerning the assets side, the fall in *insurance and pension funds guarantee schemes* fell to the benefit of the rise of *equity and funds shares*. This is mirrored by the liability side, where more traditional liabilities, *i.e. currency* and *deposit*, and *insurance and pension funds schemes*, decreased their relative weight, while *debt securities* and *equity and shares* boomed. Two elements are worth stressing. First, the FS largely financed its operation by issuing securities and funds shares. Second, the similarity of the two sides of the balance sheet is striking (see the double role of equity and shares) showing how the FS was at the same time issuer and buyer of the very same kinds of assets.

Figure 3 shows the main flows within the FS. The similarities among assets and liabilities (Fig. 2) mirror the decrease in interests (paid and received) and the rise in dividends. Shares are indeed the only kind of liability issued by the Funds. The black-dotted grey area represents the net income of the sector (i.e. the difference between outflows and inflows), which is also portrayed as a ratio to GDP by the red line. Income boomed from 2000 to around 2003. After this, we see a fall in correspondence with a sharper rise in *interest paid* relative to the corresponding increase in *interest received* (see respectively the dark grey and dotted dark grey areas in Fig. 3) until the collapse of 2008, when net income goes into negative territory. After the crisis, the net income of the FS rebounded overcoming the pre-crisis level. This was driven, first, in 2009 by *private depository institutions*, whose drop in outflows more than compensated for the decrease in gross income. The same happened, one year later, with what the flow of funds tables define as *other financial businesses*, namely the residual part of the FS after excluding the monetary authority, private depository institutions, pensions funds, and insurance companies. This portion, whose net income remained negative from 2013 to 2017, is the main driver of the post-2012 volatility of net income at the aggregate level.

In a nutshell, up to this point, the picture is the following. The FS evolved into a system in which funds and other non-traditional financial sub-sectors have become increasingly important. Interests are still the main element both as income and outflow, but distributed income, in particular dividends, has significantly risen, due to the upsurge of shares among FS assets. This transformation came hand in hand with the well-known expansion of the FS.

In our view, an obvious, although too often overlooked, part of this story is that the expansion of the financial system encompassed a surge in its level of indebtedness. While the *subprime crisis* is normally associated with impressive growth in the household sector’s liabilities, this same trend for the financial sector has been even more striking, as shown in Fig. 4.

**Fig. 5:**
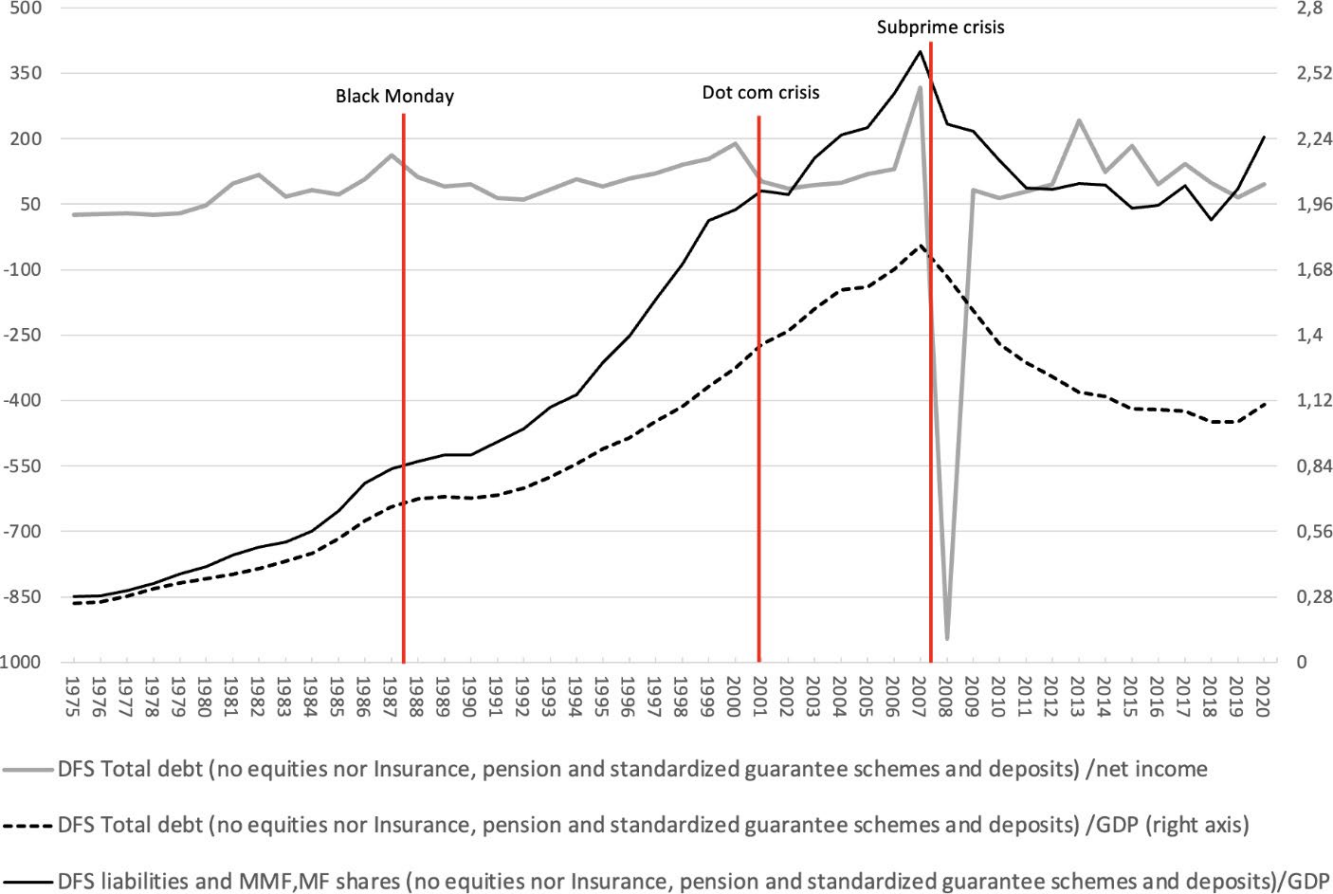
Domestic financial sector debt to net income ratio = grey line, debt (without MMF and MF shares) to GDP ratio = black dotted line; source: Financial Accounts of the United States Z1 Flow of funds, Federal Reserve Bank of St. Louis Economic Resources & Data (FRED), and author calculation

**Fig. 6:**
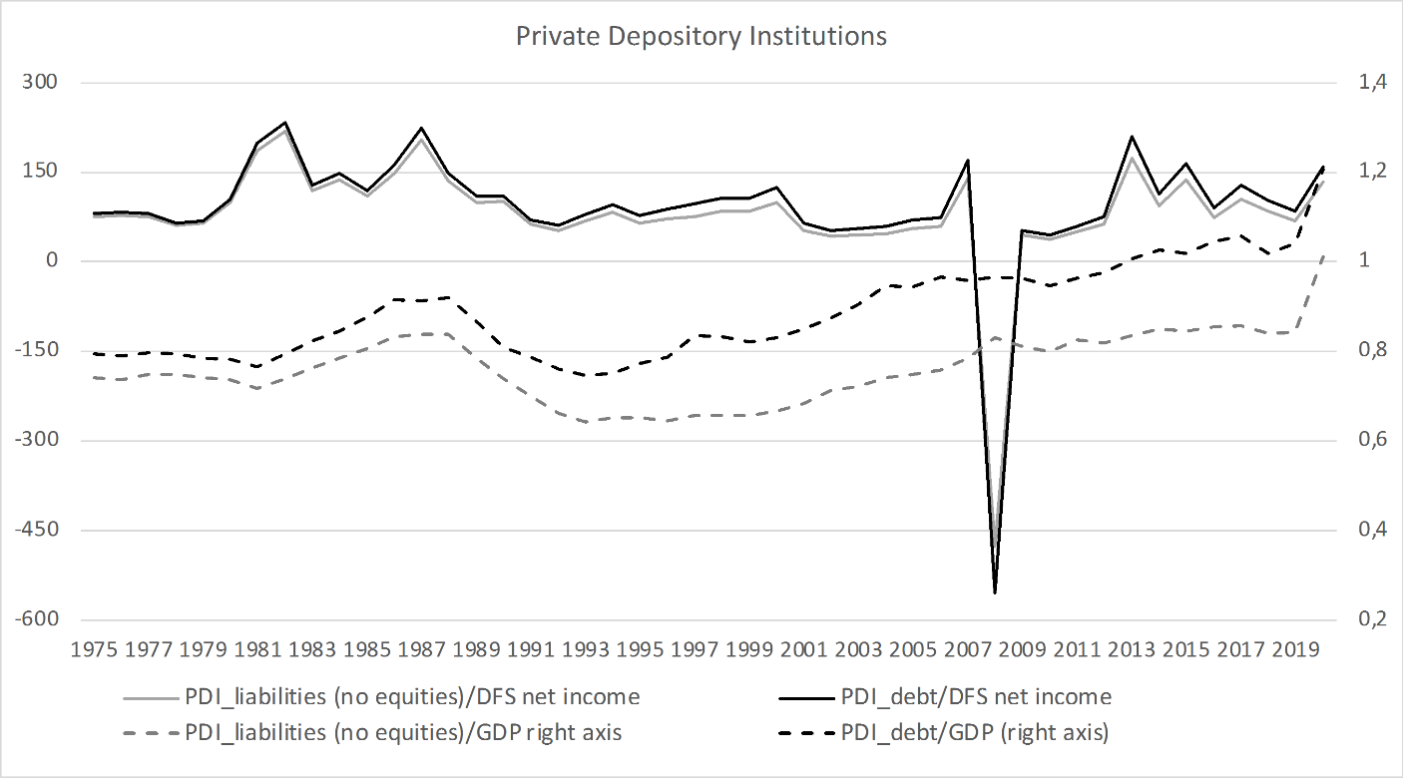
Private depository institutions debt to domestic financial sector net income ratio (grey line) and debt to GDP ratio (dotted lines); in black the ratios with respect to total debt, in grey without equities; source: Financial Accounts of the United States, Z1 Flow of funds, and author calculation

**Fig. 7:**
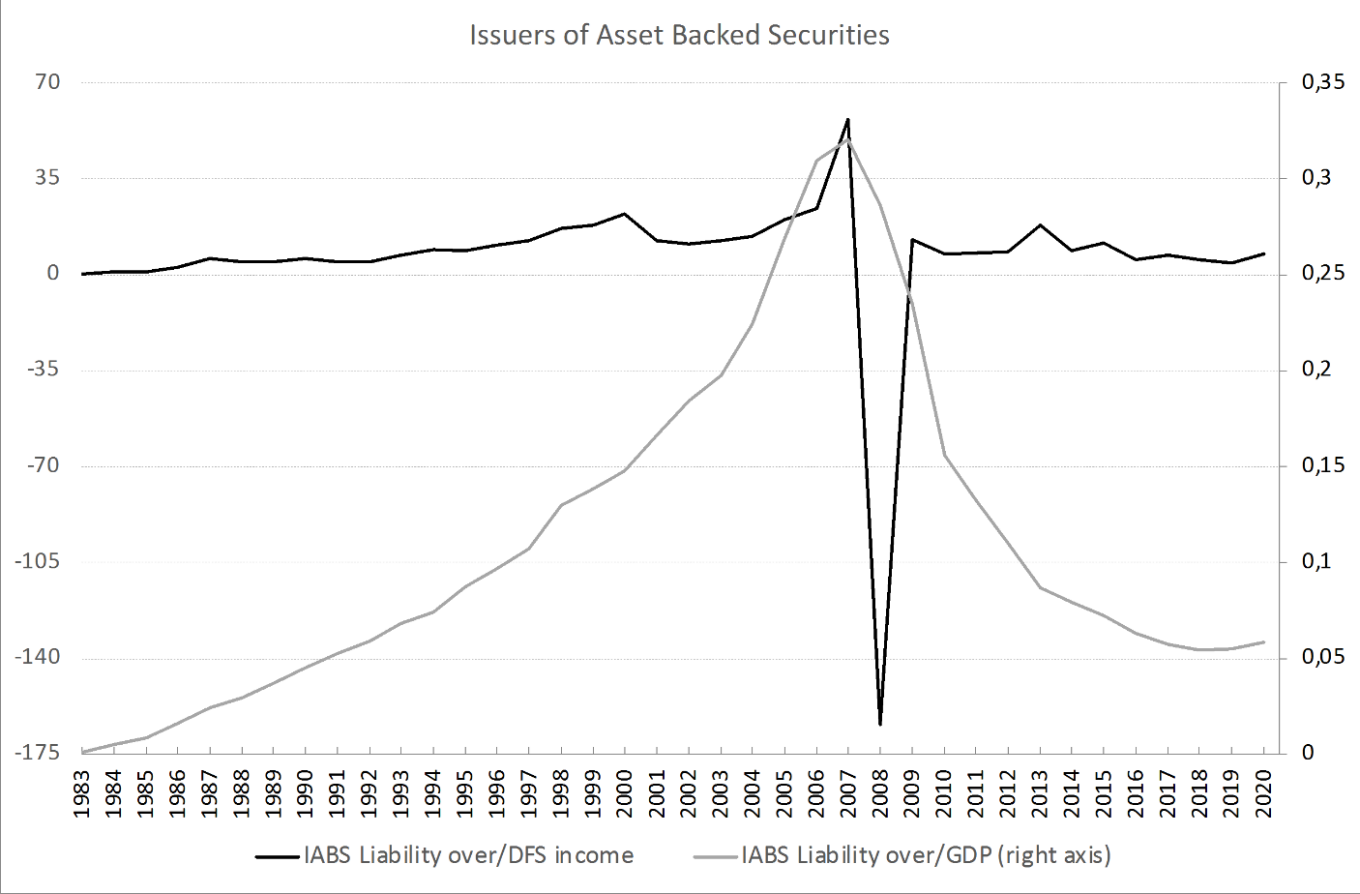
Issuers of asset-backed securities’ (IABS) debt to DFS net income ratio (black line) and debt to GDP ratio (grey dotted line); source: Financial Accounts of the United States Z1 Flow of funds, and author calculation

**Fig. 8:**
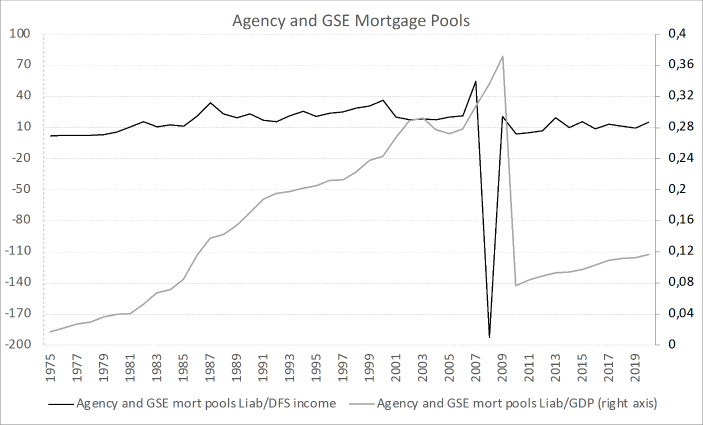
Mortgage pools debt to DFS net income ratio (black line) and debt to GDP ratio (grey dotted line); source: Financial Accounts of the United States Z1 Flow of funds, and author calculation

**Fig. 9:**
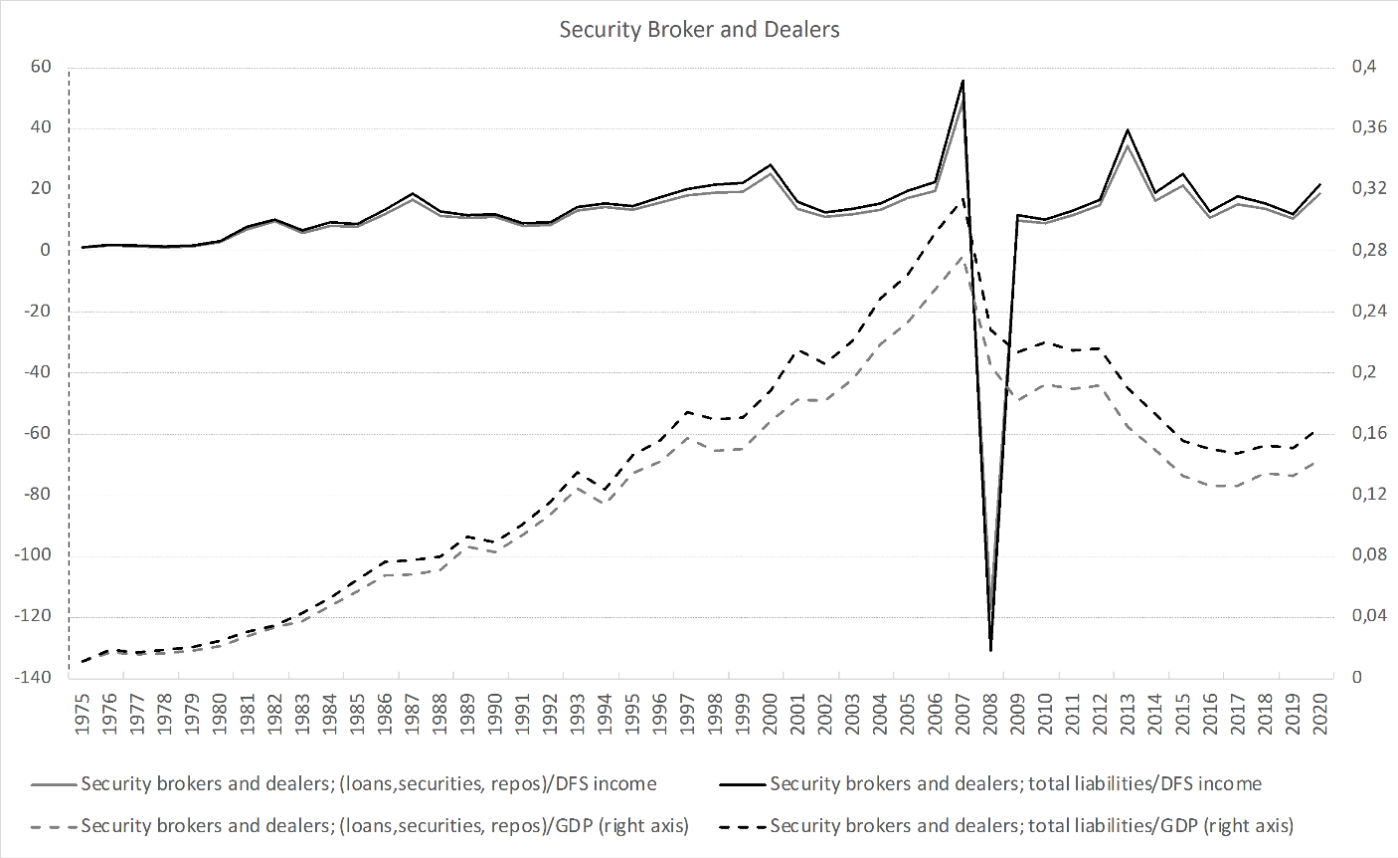
Security broker and dealer debt to domestic financial sector net income ratio (grey line) and debt to GDP ratio (dotted lines); in black the ratios with respect to total debt, in grey without equities; source: Financial Accounts of the United States Z1 Flow of funds, and author calculations

**Fig. 10:**
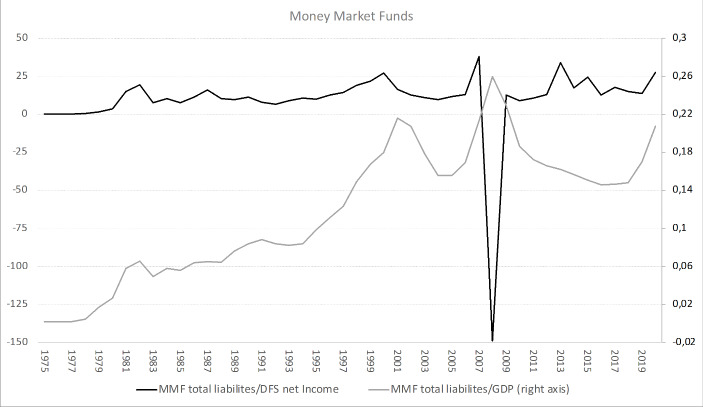
Money market funds’ debt to DFS net income ratio (black line) and debt to GDP ratio (grey dotted line); source: Financial Accounts of the United States Z1 Flow of funds, and author calculation

**Fig. 11:**
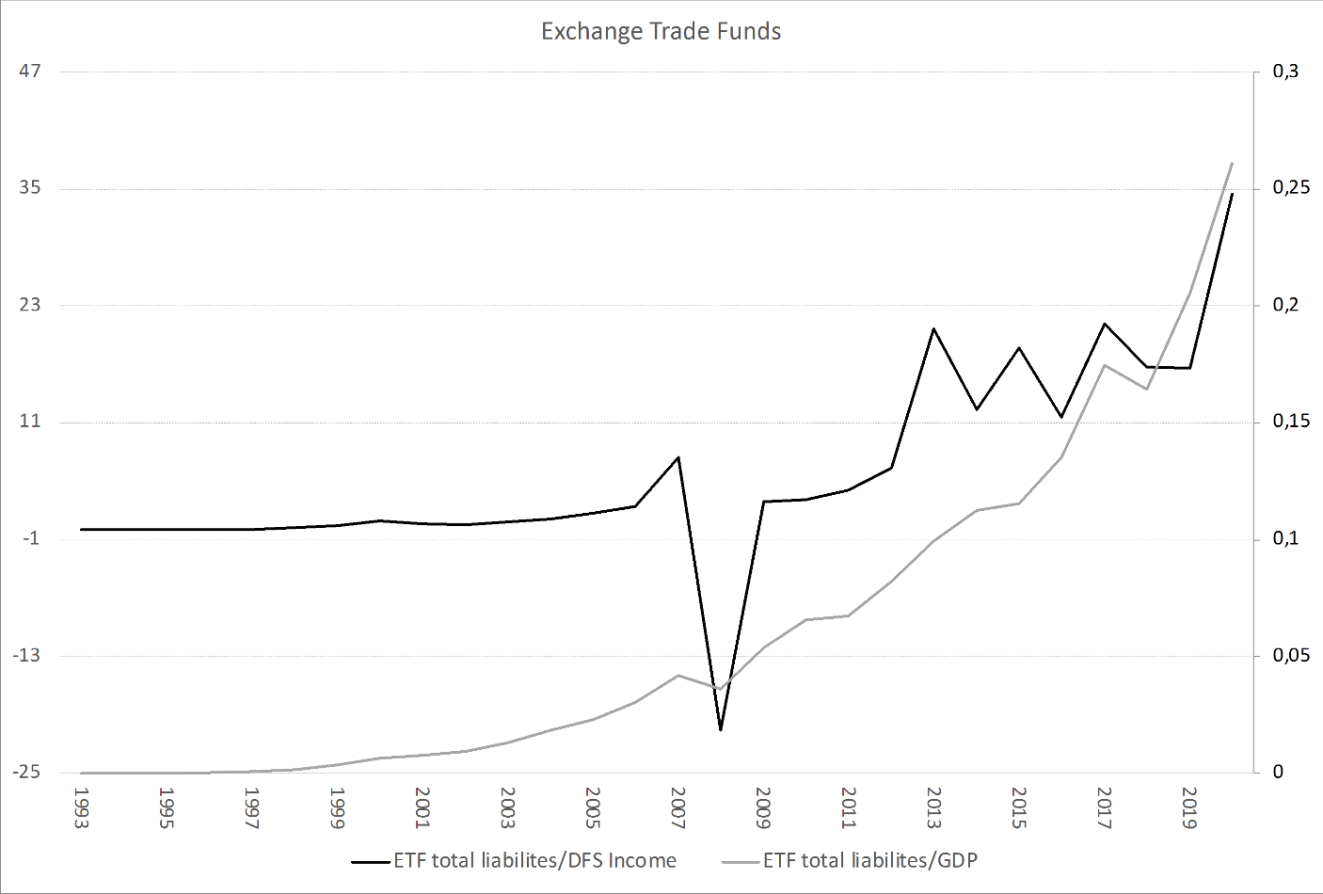
Exchange-traded funds debt to DFS net income ratio (black line) and debt to GDP ratio (grey dotted line); source: Financial Accounts of the United States Z1 Flow of funds, and author calculation

This figure compares the debt-to-GDP ratios of the main sectors of the economy: the non-financial non-corporate sector (NFNC), the non-financial corporate sector (NFC), the household sector (HH), and the domestic financial sector (DFS). Equities are not included, and for DFS two debt measures are used. The first includes securities, loans, and repos, whilst the second adds the shares. This choice is motivated by the consideration that shares do not technically constitute a kind of liability to be serviced regularly and, as in Minsky’s FIH, we assess financial fragility by looking at income and debt services - *net income*, used in Figs. 5, 6, 7, 8, 9, 10 and 11, is indeed the difference between the two. Nonetheless, as just mentioned, the various funds played a crucial role in the destabilization of the financial system, hence in the crisis. Therefore, the natural next step is to assess their relative magnitude. Moreover, in the specific and crucial case of *money market funds*, the value of a share was maintained as equal to one dollar. This rigidity, aimed at mimicking standard deposits, turned out to be unsustainable during the crisis and became a further source of instability for the sector.

To assess the implications in terms of financial fragility, we focus on stock-flow ratios. Fig. 5 shows the domestic financial sector debt over its net income (grey line) and GDP (dotted line).^12^ The ratios of DFS’ debt over net income offer intriguing results: each of its peaks from 1980 to 2010 is associated with a crisis^13^. In Minskyian terms, this could be interpreted as erosion of the *cushion of safety* (Kregel 1997) for the whole FS. A rise in the stock of debt coupled with a shrinking difference between income and debt service is indeed the quintessential feature of the upward phase of the cycle in the FIH. The indebtedness of the financial sector grew quite steadily to reach its peak in 2007, at 261% of domestic GDP.

Graphs from 6 to 11 show the same ratios for different sub-sectors within the FS. When a specific financial institution issues more than one type of liability we refer to two distinct debt measures, one including and one excluding investment funds’ shares and equity. While the data for the debts is specific to the sectors, the net income measure we use is the same across sectors, namely that of the domestic FS (since the time series at the sectoral level are not available). As the crisis determined a negative net income, from 2007 to 2009 debt to net income ratio falls in negative territory for all sectors.

Therefore, just like with the GDP, the ratio between debt and net income provides insight into the inner trends of the financial system. For sake of parsimony, besides private depository institutions, we show five of the twelve financial sectors other than pension funds, insurance companies, and monetary authority. The choice is based on the selection of the most important actors among those involved in the *securitizing system.* The traditional banking sector (*private depository institutions*) experienced the highest financial fragility in the 1980s, during the so-called *savings and loan* crisis and until the 1987 *black Monday*, after which its debt to GDP ratio dropped, to slowly recover only in the 2000s. While the banking sector played a pivotal role in the *subprime* crisis as the issuer of the loans used in the processes of securitization, other sectors within the securitizing systems carried a heavier weight in terms of financial fragility.

The sectors primarily involved in the business of securitization present the peak of their debt to FS’ income ratio in correspondence to the *subprime* mortgage crisis. The figures for *mortgage pools (MP)* and *issuers of asset-backed securities (IABS) are* extremely clear in this sense (Figs. 7 and 8). The stock of debt in both sectors boomed, growing from less than 5% to more than 30% of the GDP in three decades. The figures for these sectors show a clear pick in our proxy for financial fragility (i.e. the debt-to-income ratio) in correspondence with the crisis.

*Security brokers and dealers (SBD)*, display similar dynamics, with a steep rise in the financial fragility proxy and the debt-to-GDP ratio (Fig. 9). However, while our fragility proxy shows some volatility after the crisis, the fall in debt-to-GDP has been less dramatic than for MPs and IABS.

Finally, the trends for *Money market funds (MMF)* and *Exchange-traded funds (ETF)* are shown in Figs. 10 and 11. Unlike Graph 1, we decided to separate the different kinds of funds, because their behaviour appears rather distinct, in particular after the crisis. While MMF’s graph resembles that for SBDs (except for a higher role in the 2001 financial crisis and a steeper rise of both measures in the most recent years), ETFs completely differ, as both fragility and debt-to-GDP show a dramatic increase in the last decades. The graphs of all the sectors involved in the securitizing system portray very high debt-to-GDP ratios (between 26% of MMF to 37% of mortgage pools).

The (disaggregated) data presented above confirm a deep transformation in the financial sectors, with new actors increasing their size and influence both within the sector and in relation to the economy. We highlighted the rising indebtedness of the financial sector and its increased financial fragility in correspondence with crises. In particular, the data confirm how several actors within the financial system were involved in the securitization business. The resulting increased indebtedness in their balance sheet did not take place exclusively through an accumulation of securities and shares, but also repos and loans (especially in the case of *security brokers and dealers* and *real estate investment trusts*).

This dynamic brought the system towards unsustainable financial fragility. Following the Federal Reserve Bank of St. Louis’ Financial Crisis Timeline^14^, the first sign of worsening conditions was a press release at the end of February 2007 in which the Federal Home Loan Mortgage Corporation (Freddie Mac) announced the end of acquisitions of the riskiest *subprime* mortgages and mortgage-related securities. On the 2nd of April, New Century Financial Corporation, a real estate investment trust and leading US independent *subprime* mortgage lender filed for bankruptcy protection. Four months later, another real estate investment trust, the American Home Mortgage Investment Corporation, asked for the same bankruptcy protection. Almost at the same time, the investment bank Bear Stearns liquidated two hedge funds that invested in various types of mortgage-backed securities. Real estate investment trusts inaugurated the collapse. The repo and the securitization systems at the basis of the *subprime* lending followed immediately after.

Our empirical assessment is a starting point in understanding the transformations within the financial sector and its intensifying fragility. To this end, we employed the relative movement of the income and debt of the financial sector as the key Minsky-inspired descriptive measure. This structural evolution entails variations in the target portfolios’ composition, causing fluctuations in the financial sectors’ demand for various types of financial assets, hence affecting assets’ prices and ultimately fuelling boom and bust cycles.^15^

## Financial firms and financial production

Section 2 showed how it is possible to contextualize financial innovations by synthesizing Schumpeterian and Minskyian views about the *‘entrepreneurial’* and *‘innovative’* aspects of the financial sector. Section 3 provided a picture of the environment within which these features could flourish, thus fuelling the endogenous forces at the basis of the GFC. The financial system was at the same time issuer and buyer of the very same kinds of assets. In addition, the empirical evidence strongly suggests that to disentangle the endogenous dynamic of financial fragility, the focus of the analysis should be on specific sectors within the securitizing system rather than on the ‘traditional’ banking sector. The indebtedness of these sectors (see Figs. 7, 8, 9 and 10) grew at a higher pace than the income of the financial sector, hampering their abilities to meet their financial obligations. Today’s financial system “bears little resemblance to that of our parents’ generation. […] Technology has transformed the efficiency, speed, and complexity of financial instruments and transactions” (The Financial Crisis Inquiry Commission, 2011, xvii). In our view, any effort aimed at understanding the impact of the financial system on the economy cannot but overcome the view of financial institutions as simple intermediaries. As such, the 2007-8 crisis should not simply be viewed as the result of the over-indebtedness of a part of the household sector, receiving loans from financial institutions intermediating the saving of the wealthier household.

As underlined (among others) by Gorton (2010), the roots of the crisis, are to be found in innovation within the financial system itself, namely *securitization* and, we argue, in the new roles assumed by the different sectors within the financial system. Securitization indeed was not just a mere evolution of financial intermediation. It created a new business which reshaped the financial realm and its relations with the real sector. Recalling the main features of this innovation also by referring to the sectors and the data presented in Section 3 may help better appreciate its impacts.

Innovation can take multiple forms (Winter 2006). In the last three decades, rather than in terms of organizational structure, the banking sector went through technological changes in the form of new services and products (Fram and White 2014). Securitization is “the business of packaging and reselling loans, with *repo* agreements as the main source of funds” (Gorton and Metrick 2012:425). It allows the transformation of illiquid loans into liquid financial securities. The process is rather complex, encompasses several steps, and, directly or indirectly, involves all the financial sector entities. Private depository institutions (traditional banks) issue loans, mainly mortgages, which are moved into the balance sheets of Special Purpose Vehicles. These, in the data of the Flow of Funds, are *issuers of asset-backed securities*, and, in the case of mortgages issued by government agencies and *GSE, mortgage pools*. Loans are then merged into mortgage pools and differentiated according to their riskiness, hence transformed into securities, and then sold to the financial markets (*security brokers and dealers)* and funds (as *money market funds).*

We believe that it is revealing to look at this complex process as a ‘Fordist mass-production industrial model’ (Goldstein and Feldstein, 2017), which entails four steps: (a) credit creation, i.e. the issuance of the loans, (b) its transformation into a financial commodity through standardisation and partition, (c) the transformation of the financial commodity into complex financial products, and (d) the sale of the structured asset to other financial institutions.

*Credit creation* exerts its effects both in the real and in the financial system. On the real side, money entered the economy providing the borrower with the purchasing power needed, say, to buy a house (in the case of MBS). The money spent entered the economic system and could ultimately be saved and allocated in the financial market, expanding the liability side of financial institutions’ balance sheets, while potentially feeding the demand for securitised loans. On the financial side, it created the credit relation embodying the financial *raw material* then transformed into a financial commodity to be used for financial production. The core sector for this stage was *Private depository institutions* (Fig. 6) flanked by other non-bank credit issuer sectors such as *Real Estate Investment Trusts*.

In the second stage, loans are pooled together and then divided into tranches typically with different risk exposures. This, which is the first step of the securitization process (see Pozsar et al. 2013) can, in our view, be conceived as a process of transformation of credit relations into *financial commodities* (Caverzasi et al. 2019)^16^: debts are indeed partitioned and standardized thus transformed into financial inputs to produce structured financial assets. Therefore, the new banking scheme ‘originate and distribute’ can also be conceived as the first step of a generalised process of *commodification of financial relationships* (Botta et al. 2015). *Commodification* had the twofold role of cleaning the balance sheet of a credit issuer while creating a standardized input for a production process. *Mortgage pools* and *issuers of asset-backed securities* (Figs. 7 and 8) implemented this step.

The *production of financial assets* by financial firms is the third phase, which is carried out similarly to what happens in a non-financial production process. Acquired inputs (the raw materials) or ‘primary’ financial assets or liabilities such as mortgages are used to produce structured financial instruments. This type of production.

“is essentially analogous to the manufacturing firm where one production department produces and supplies an output which is used directly as an input in another process. Eventually, the intermediate outputs culminate in the final economic output of the firm, i.e. earning assets. The output of the financial firm is, therefore, produced with capital, labour, material, and loanable fund inputs where loanable funds are ‘produced’ through other production operations of the financial firm.” (Sealey and Lindley, 1977:1254).

Financial firms used securitized mortgages (MBS) as inputs to produce CDOs, a structured financial product that pools and repackages cash-flow generating assets (the collateral). Complex securities were made of tranches with different seniorities from various loans. Earning assets are the output of the financial production process that, as happens for traditional businesses, generates products that are more highly valued in the market than the inputs used to manufacture them. Securitization granted both liquidity and credit enhancement (Pozsar et al. 2013). Illiquid long-term assets, such as mortgages, were transformed into more liquid securities, while priority claims allow obtaining safe assets from more senior tranches, often with the certification of a credit agency. Financial engineering added further steps to this chain of production, creating increasingly complex financial instruments, like ‘squared CDOs’ or ‘synthetic CDOs’. This structure, also thanks to its obscurity, made the products appear safe, hence obtaining the notorious ‘AAA’ ratings.^17^ The main player in this stage were *security brokers and dealers* (Fig. 9).

In the last stage, through the intermediation of funds like *Money market funds* (Fig. 10) and *Exchange-traded funds* (Fig. 11), the assets were sold to an eager market, in which demand was driven by several factors, as we will show in the next section. The boom in the production of financial assets is well described by the words of the executive director at Morgan Stanley: “We almost couldn’t *produce* enough to keep the appetite of our investors happy. More people wanted bonds than we could actually *produce*. That was our difficult task, was trying to *produce* enough” (cited in Goda and Lysandrou, 2014:314, italics added).

This spectacular growth needed to be financed, and the types of liabilities issued to finance this new phase differed among sectors. *MMF* (just like the other funds), on the one hand, and *issuers of asset-backed securities* and *mortgage pools*, on the other hand, had their unique type of liability, respectively shares and securities. *Security brokers and dealers*, just like *real estate investment trusts* and *other financial businesses*^18^, mainly financed their business through loans and repos. As discussed in Section 3, these sectors became increasingly indebted until the revenues from their assets became insufficient to service their debt. The *subprime crisis* ought to be seen as a case of (financial) productions increasingly financed through debt.

While non-bank financial institutions embodied the novel ‘entrepreneur’, the traditional banking sector played the double role of credit and (financial) commodity provider. Indeed, although most of the financing through repurchase agreements (repos) took place within the investment banking sector, commercial banks represented the main external source of funds (see Caverzasi et al. 2019).

*Repos* (a form of ‘collateralised loans’) had a crucial role in this scheme, being both a safe source of finance and a very liquid form of short-term investment and a source of demand since MBS were used as collaterals. As mentioned, the whole financial system was involved: either in one of the steps of this financial *supply chain*, which started from the creation of the loans and ended with the manufacturing of complex financial products, or in the demand side (Botta et al. 2015). Also, highly regulated institutional investors such as pension funds had a large part of their portfolio (around 19% just before the crisis) invested in shares of funds, which in turn heavily invested in securitised products. At the same time, from the 1990s to the crisis, *private depository institutions* steadily invested more than 10% of their assets in agency- and GSE-backed securities.

A word of caution must be mentioned. While the distinction of different financial institutions’ roles in the securitizing system is important for analytical purposes, the boundaries are not so neat in practice. As underlined by Goldstein and Fligstein (2017), financial institutions pursued strategic vertical integration appropriating profits in all layers of the mortgage industry. Therefore, they were able to implement all the different steps of the securitization process within the same institution or among affiliates.

*Investment banks* and *security brokers and dealers* can certainly be identified as major manufacturers of these financial products. However, the evolution of the US financial system from the weakening and demise of the Glass-Steagall Act to the flourishing of holdings and the wide interconnection among diverse financial institutions made the distinction between ‘financial entrepreneurs’ and ‘credit providers’ only conceptual, while witnessing the presence of almost fictitious counterparts.^19^ This feature made the credit provision mechanism explosive. The financial sectors involved in the securitizing system boomed, with their size relative to the rest of the financial system quadrupling in two decades.

Although the similarities between traditional and financial firms are very significant, the differences are non-trivial. While the former experiences constraints on potential utilisation from physical limits posed by technology, the latter deal with the less-constrained production of intangible financial products (Nightingale and Poll 2000).^20^ Theoretically, financial firms’ production has no limitations, apart from the ‘scarcity’ of creditworthiness inherent to the system. Banks, while endogenously creating money through loans, at the same time create the (raw) commodity essential to the financial production process. This determines a condition of *endogenous creation of commodity*, a unique privilege of the financial industry, for which a counterpart in the realm of traditional businesses cannot be found. Moreover, even the constraint posed by the availability of creditworthy borrowers has been relaxed due to securitization. Banks are now able to clean their balance sheet from risky assets, whenever these are moved to the balance sheets of SPVs, transformed into structured financial assets, and then sold. This removes what in Minsky’s view represented the real limit to credit supply, namely the borrower’s risk (Minsky, 1986).

The rupture of the legal boundaries between credit providers (commercial banks) and financial producers (investment banks), together with the weakening of the limits posed by lenders’ risk, set the stage for the explosive dynamic we witnessed: “Securitization was one of the most brilliant financial innovations of the 20th century.” (The Financial Crisis Inquiry Commission, 2011:10). Moreover, as mentioned above, credit creation also implies the creation of purchasing power. Loans create deposits that disappear once the loan is repaid or when deposits are used to purchase securitised loans (see Botta et al. 2015). It is exactly this self-feeding element that explains what Borio and Disyatat (2010) labelled as the ‘excessive elasticity’ of the financial system.

## A structural reading of the crisis

In a similar way to what explains entrepreneurs’ appetite for innovation, the opportunity for an increase in profitability for the whole financial sector has been the key driver for financial innovation like securitization (Botta et al. 2020). On the one hand, the process entails the debated passage from the ‘originate to hold’ to the ‘originate to distribute’ banking model (Bord and Santos, 2012) and, on the other hand, the evolution of part of the financial system into a system of production of financial assets. We dubbed the entities involved in the production process ‘financial firms’.

Once the entrepreneurial innovative feature of financial institutions is acknowledged, we can look at the GFC through Minsky’s financial-Keynesianism and suggest an updated version of the FIH within a broader macro-financial perspective. This updated FIH appears to be dual. The destabilising dynamic in the building up of the recent crisis was not linked to the indebtedness of traditional firms and took place among households and ‘financial firms’. We argue that (i) the indebtedness of the former was largely due to the activities of the latter, and (ii) the financial position of the latter, usually overlooked by the literature, was equally important to the widely discussed household debt.

The first element is the massive demand for securities that fuelled the process. The spectacular rise of the US financial sector starting in the 80s is a well-known stylized fact. While the *dot.com* crisis reduced the role of equities, the relative growth of the financial sector with respect to the real side of the economy did not stop until the *subprime* crisis (see Fig. 1). Therefore, after 2001 we witnessed a general portfolio reallocation from equities to securities. Going back to our introduction, this is where the stock of savings comes into play, with its allocation bound to determine changes in the relative price of assets, and thus start the booming phases of the securitizing system. However, is the creation of new liabilities (thus assets) with the creation of credit that determined the rise in the debt level of the economy toward unsustainable levels. We believe that the creation of credit through the issuance of mortgages was largely driven by the demand from financial firms. Their demand for inputs (financial commodities) for the financial production process was in turn driven by the demand for securities. The possibility for banks to get rid of loans through securitization removed the constraint posed by borrowers’ creditworthiness. This boosted the supply of primary financial commodities and made banks able to meet a rising level of demand.

Next to the portfolio reallocation, further elements concurred in the determination of a high level of demand for securities. In a situation in which the financial side of the economy was growing faster than the real one, the tendency of the financial system to have a rather stable share (i.e., one-third) of safe assets (Gorton, 2012) implied the necessity of holding an increasing amount of privately produced safe asset (ABM, MBS, etc.). Moreover, other evolutions of the financial system strengthened the hunger for securities. Repos, which require the presence of safe assets as collateral (Gabor 2016), became increasingly popular. Money market funds experienced spectacular growth and were largely investing in securities (see Section 3). This is linked to the aforementioned two-fold impact of inequality. On the one hand, this determined a demand for assets by the better-off (usually intermediated by funds) and, on the other hand, a demand for credit by the worst-off. While these elements explain an increase in the demand for securities, the low yields on safe Treasury Bonds in the year preceding the crisis made other forms of fixed-income investment more attractive.

The production of securities to match this rising demand needed an increasing amount of inputs: banks needed to issue credit. We saw how the new ‘originate to distribute’ facilitated supply, but what about the demand side? It is worth reminding that the main reason behind households’ indebtedness, especially in the US, is the purchase of a dwelling. The peculiar environment of the US market from 1996 to 2005 saw houses price rise by 45%, an increase not driven or explained by fundamental factors such as income or population growth rates (Baker 2010). Within this context, securitization allowed banks, and the financial system, to leave their ‘usual’ position in which lending is hedged against default, and in which the borrower could always pay back both interest and principal through mortgage repayments. Banks encouraged lending also by altering the risk assessment ratio used in the decision for conceding a loan: instead of the ‘mortgage-to-income’ ratio, they started employing the ‘loan-to-value’ ratio, where the denominator indicates the appraised value of the asset (i.e. dwellings), thus artificially expanding the demand by ‘credit worthy’ borrowers. Furthermore, it is easy to see that the amount lent will be higher in a situation of apparently everlasting increase in the price of houses.^21^ In addition, increasing house prices meant larger mortgages. In the context of sluggish household income (Cynamon and Fazzari, 2008), substituting the mortgage-to-income ratio with the loan-to-value one proved to be a real boost to banks’ lending. This fuelled the emergence of a perverse dynamic in which, on the one side, the banking system was pushing lending, and, on the other side, households’ demand for borrowing was increasing.^22^

In a scenario with 1) financial markets eager for securities and 2) an evolved financial system capable to manufacture securities starting from an input that it was itself generating (i.e., credit), financial fragility grew on two parallel roads. On the one hand, households got indebted, obtaining easy credit, and fed the house price boom. On the other hand, financial firms, eager to participate in the profit-generating process of producing and selling securities, issued increasing levels of debt to finance their activities thus feeding the boom in the market for securitised loans.

The macroeconomic implications of the link between money, debt, and investment highlighted by Skidelsky (2009), assumed a peculiar form. The banking system was at the very centre of the securitization scheme. It supplied liquidity in form of *repo* agreements to financial firms (i.e., brokers and dealers and investment banks), which in turn used these funds to purchase ABS (e.g., MBS, home equity loans) previously assembled by the banking system itself (also including special purpose vehicles). Financial firms obtained access to the *repo* using ABS held as collateral. This clarifies how banks have been able to stimulate the demand for these securities. Money entered the economy through mortgages, with limited benefit to the productive capacity of the system. The two assets created in the process were dwellings, whose construction may stimulate economic growth just in the short run, and financial assets, which are ultimately debt. What appears to be extraordinary in this period is not an exceptionally low level of real investment.^23^ It is therefore hard to maintain that the causal link may run from the real to the financial sector, while the debt level of households and the financial sector stand out (as shown in Section 3).

The business was at first both remunerative and secure, a booming dynamic kicked in both in the real estate and the securities market. The concept of *pseudo-wealth*, namely ‘wealth that individuals perceive they have, but which is to some extent divorced from the physical assets that exist in society’ (Guzman and Stiglitz 2021:372), perfectly captures the essence of what was taking place. The household sector was taking advantage of a supposed ever-increasing value of houses, while the financial system’s perception of the value of what they were manufacturing, holding, and using as collateral, was driven by a ‘false sense of security’. Both the booming dynamics in the real estate and the securities market were indeed built on debt. The productive capacity of the system (i.e., real investment) was not directly involved, so the increase in debt was not matched by an increase in the ability to service it.

The twin financial fragilities mounting on households and financial firms’ balance sheets can be interpreted through the lens of the FIH. We will focus on the financial markets as Minskyian interpretations of the real estate side of the story have already been put forward.

The evolution of the business appeared to be stable at first, with financial firms playing the role of *hedge* units. Exactly as Minsky described in the FIH, the *destabilizing stability* then exerted its effects, also thanks to the deregulation of financial markets (Sherman, 2009). In the context of rising house prices and sustained economic growth, the credit provision mechanism flourished, and asset manufacturing boomed. The optimism made all the actors involved less risk-averse. Borrowers and lenders in the credit market, issuers of asset-backed securities, financial firms, and financial intermediaries, all assumed increasingly speculative positions. Therefore, the rise in instability marched on two legs. First, the quality of financial commodities and financial assets fell dramatically. The issuers of the loans significantly lowered their credit standard. The average Loan-to-Value ratio reached its peak of 94% in 2005 (Duca et al. 2011) and the *sub-prime* mortgages, which used to represent an exception − 8% of total mortgages in 2003 - became almost consuetudinary, reaching the climax of one-fifth of newly originated mortgages in 2005 and 2006.^24^ Meanwhile, the success of CDOs led financial firms to issue increasingly riskier financial assets. CDOs based on *subprime* collateral passed from representing 5% of total CDOs issued in 2000 to 36% in 2007 (Barnett-Hart 2009). Second, financial firms (banks, investment banks, and hedge funds) dramatically increased their leverage and invested in MBS (Greenlaw et al. 2008). The dynamics portrayed in Section 3, showing the debt levels growing much faster than the income of the financial sector, embody the switching point from *hedge* to *speculative* and eventually *Ponzi* positions, in which ‘success breeds excess’ (Minsky, 1992). Additional evidence of this can be found in the chain of repledgement of collaterals in the *repo* market, which Singh (2011) shows to have peaked before the crisis, and in the skyrocketing increase in the number of *repo* deals by investment banks. *Ponzi* positions are not sustainable in the long period, and the bubble inevitably had to burst.

When households started to default and the financial commodity showed to be less safe than perceived, financial firms proved unable to meet their financial commitments, starting to default themselves, and the crisis spread through the interrelated balance sheets of the financial sector. The first to be hit were those issuers specialized in mortgages, that is *real investment trusts* such as New Century Financial Corporation – which filed for bankruptcy in April 2007, and from there all the financial firms that got indebted to be part of that business.

The new ‘location’ of the financialized FIH brings aspects of novelty in its consequences.

Financial firms panicked and started selling their assets (securities) whose values collapsed, as in the first stages of the standard Fisherian debt-deflation story retrieved by the FIH.^25^ This time, however, the ‘fire sale’ of assets affected mainly securities. From being the justification for strong credit ratings, the obscurity of structured securities became a motive for panic, due to the impossibility of pricing the assets.^26^ This situation led to a fire sale of securities taking the form of a ‘run on *repo*’. The strong increase in *repos* haircut showed by Gorton and Metrick (2012), which is the amount of collateral demanded (thus measuring the supposed underlying risk of the collateral) mirrored a decrease in the valuation of the asset compared to its market value: “With declining asset values and increasing haircuts, the US banking system was insolvent for the first time since the Great Depression” (*ibid.*, 2012:425). The second ‘run on *repo*’, with a 20% points increase in the haircut in just one month, occurred in September 2008, when Lehman Brothers was declared bankrupt: “In this second event, we see parallels to 19th-century banking crises, with a famine of liquidity leading to significant *premia* on even the safest of assets” (Gorton and Metrick 2012:448). These fire sales of securities and their subsequent fall in price represent nothing more than the last step of the FIH, i.e. the start of a Fisherian debt-deflation.

While in the original FIH, financial fragility surged within the financing of real investment, in this updated dual version, the kind of investments involved were either dwellings or financial instruments (i.e., debt), both having only a limited impact on the overall productive capacity. From a macro perspective, the essence of this dynamic was financially unsustainable. As someone’s financial assets are someone else’s liability, at the aggregate level, an economy investing in domestic financial assets is an economy investing in its own debt.

## Conclusion

The debate about the causes and the mechanisms of the 2007-8 crisis is still ongoing. This paper explains the financial crisis by drawing on a financial reading of Schumpeter’s and Minsky’s contributions, in particular using an adapted version of Minsky’s financial instability hypothesis that is consistent with the key features of a *financialized monetary theory of production*. Our empirical evidence suggests a significant transformation in the US financial sector from the late 1970s, characterized by rising indebtedness and financial fragility. The evolution of US financial institutions led them to transcend their traditional role as credit providers and become ‘financial producers’ through securitization. The coincidence of this double role within the same entities made the dynamic in the credit provisioning system explosive. We use this to provide a novel explanation of the financial crisis. Our analysis aims at providing a ‘structural interpretation’ of the crisis, identifying and understanding the endogenous forces that progressively drove the US economy towards an unsustainable financial position, and made the crisis an inescapable event. The pathogens that led to the crisis were inherent in the specific innovative form that US capitalism assumed since the early 1980s. The crisis erupted in the *subprime* mortgage market precisely because of the specific forces driven by the new role that financial firms acquired through securitization.

Minsky captured the *money manager capitalism* phase in the evolution of capitalism and was able to grasp the cornerstone role played by securitization. This innovation found its fuel within what was believed to be one of the most stable components of the economic system, namely the real estate market. We argued that the traditional ‘financialization’ and ‘money managers capitalism’ literature is insufficient to understand the unfolding of the crisis. These theories do not give a central role to financial production and see household and business indebtedness as a triggering, rather than functional, element in this story. Our interpretation of the crisis can be summarized as the description of a ‘financial demand-driven macro-dynamics’. The voracious demand for securities by the actors involved in the securitization process triggered an explosive production of structured financial products, for which the condition of chronic indebtedness of the household sector was the fundamental raw material. The unsustainable dynamic primarily sprang from the behaviour of financial firms. The trends in the household sector appear as a reverberation of more profound destabilizing dynamics that were inherent to the financial sector itself. As banks are the endogenous creator of money, financial firms became endogenous manufacturers of financial assets, with their balance-sheet management activities exemplifying a process of financial production.

Our empirical analysis confirms that the financial sector is characterized by continuous evolution. The relevance of these transformations for the understanding of future tendencies is embodied in the post-2010 volatility. In particular, the extent to which the phenomenal rise in the financial fragility of Exchange Traded Funds (which comprises the same real estate investment trusts who initiated the domino effect of the GFC) will have an effect at the macroeconomic level remains to be seen. Our analysis should be considered as a starting point, which calls for a more detailed and robust empirical analysis.

The major interventions of the Central banks after the crisis, *in primis*, but also their reactions to the COVID-19 pandemic and the war in Ukraine, go hand in hand with the evolution of financial entities, which always try to overcome their regulatory and productive limits. This paper calls for future research to gain a deeper understanding of the active and innovative role of financial firms within developed capitalist systems.

## Data Availability

The data that support the findings of this study are available from the Financial Accounts of the United States - Z.1 available at https://www.federalreserve.gov/releases/z1/default.htm.

## Footnotes

1 Our condensed review cannot fully reflect the richness of all the explanations of the GFC. This general detour helps us highlight and contextualise the novelty of our contribution. Goda (2017) provides a comprehensive analysis of the different crisis theories within the neo-classical, new-Keynesian, Marxian, and post-Keynesian traditions. Sufi and Taylor (2021) surveyed the recent mainstream literature on the financial crisis.

2 Van Treeck (2014) provides a survey of the literature about the inequality-crisis nexus, discussing the evidence about inter-household inequality as a key element in the decrease in savings, the rise in private debt, and the credit bubble.

3 Highly telling is Lucas (2009) on the inability of the workhorse model of this literature in foreseeing the crisis since it can only produce “a forecast of what could be expected conditional on a crisis not occurring”.

4 A clarification of “productive” assets is missing in Skidelsky’s explanation. This is rather important since houses are assets too.

5 On this point see also Caverzasi et al. (2019).

6 See also Bezemer (2014).

7 See Table 1 in Perez (2011:190) but also Freeman (1995).

8 In particular, the shift from the first (productive) wave of finance to the second (speculative) wave evokes the passage from *hedge* to *speculative* finance in Minsky’s taxonomy.

9 The elements reported here are not exhaustive. For a thorough theoretical analysis of Minsky’s work see Bellofiore and Ferri (2001) and Papadimitriou and Wray (1998) among others.

10 This is largely in line with the literature on shareholder-value orientation (Lazonick and O’Sullivan 2000).

11 For sake of clarity, we condensed the 18 sectors in which the US flow of funds divides the US financial system into 12 sectors, grouping the pension funds and insurance companies on the one hand, and the different kinds of funds on the other hand.

12 According to the FIH, the fragility of the economy is due to the rising number of *Ponzi* firms, coupled with balance sheets interrelations. It is therefore tricky to apply this perspective to macro data since at this level the dynamics are not necessarily noticeable (Lavoie and Seccareccia 2001).

13 The peak in 1982–1983 is matched by the early 1980s recession too. We do not to include this in the graph given the difficulties in delineating the chronological location of the financial trends.

14 Available at https://fraser.stlouisfed.org/timeline/financial-crisis (last accessed on 16.01.2023).

15 A follow-up investigation could be based on an econometric analysis exploring how the composition of the financial system drives the price dynamics and its effects on the economic system at large.

16 See also Lysandrou (2005) on this specific point.

17 As for example in the infamous fraud case of ‘Abacus’ by Goldman Sachs. See for example this article available at: https://www.theguardian.com/business/2010/jul/16/goldman-sachs-record-abacus-fine for a summary of this case (last accessed on 15.01.2023).

18 Not portrayed in Section 3 because smaller in size and because of breaks in the series respectively after Q2 2013, due to the consolidation of off-balance sheet holding, and Q1 2009 due to the transition into holding companies.

19 From a legal point of view, further disaggregation within the non-depositary institutions is evident (Gorton and Metrick, 2010). However, after different waves of financial deregulation, these distinctions became blurred.

20 In addition to ‘physical constraints’, ‘temporal constraints’ are almost inexistent in financial firms. In fact, the cycle of financial production can be extremely short. The key phases of the process (i.e. 1. the collection of liquidity through repos, 2. the purchase of financial commodities, 3. the production of derivatives, 4. the sale of financial products, 5. the final repayment of the repos) can all take place in twenty-four hours.

21 Qi and Yang (2009) discuss the importance of this new ratio in banks’ lending showing how current loan-to-value has been the most important positive determinant of the size of banks’ losses when the borrower defaulted on loans.

22 Furthermore, banks became less strict about screening potential borrowers, which has been incentivized specifically by securitization practices (Keys et al. 2010).

23 According to data from the U.S. Bureau of Economic Analysis, the ratio of gross private domestic investment to GDP was not particularly low, on average 19.44% from 1995q1 to 2000q1 and 19.54% from 2002q1 to 2007q1. The picture does not change when excluding residential investment.

24 See Harvard Joint Center for Housing Studies (2008).

25 A cycle of falling prices and rising defaults caused by excessive debt and monetary contraction (Fisher 1933).

26 On the 9th of August 2007, BNP Paribas suspended the calculation of the Net Asset Value of major investment funds, given the “complete evaporation of liquidity in certain market segments of the US securitisation market”. Full statement here: https://group.bnpparibas/en/press-release/bnp-paribas-investment-partners-temporaly-suspends-calculation-net-asset-funds-parvest-dynamic-abs-bnp-paribas-abs-euribor-bnp-paribas-abs-eonia (last accessed 16.01.2023).
